# Evaluation of purity with its uncertainty value in high purity lead stick by conventional and electro-gravimetric methods

**DOI:** 10.1186/1752-153X-7-108

**Published:** 2013-06-26

**Authors:** Nahar Singh, Niranjan Singh, S Swarupa Tripathy, Daya Soni, Khem Singh, Prabhat K Gupta

**Affiliations:** 1 Analytical Chemistry Division, CSIR- National Physical Laboratory, Dr K. S. Krishnan Marg, New Delhi 110012, India

**Keywords:** Lead, Conventional gravimetric, Electro gravimetric, Buoyancy, Uncertainty evaluation

## Abstract

**Background:**

A conventional gravimetry and electro-gravimetry study has been carried out for the precise and accurate purity determination of lead (Pb) in high purity lead stick and for preparation of reference standard. Reference materials are standards containing a known amount of an analyte and provide a reference value to determine unknown concentrations or to calibrate analytical instruments. A stock solution of approximate 2 kg has been prepared after dissolving approximate 2 g of Pb stick in 5% ultra pure nitric acid. From the stock solution five replicates of approximate 50 g have been taken for determination of purity by each method. The Pb has been determined as PbSO_4_ by conventional gravimetry, as PbO_2_ by electro gravimetry. The percentage purity of the metallic Pb was calculated accordingly from PbSO_4_ and PbO_2._

**Results:**

On the basis of experimental observations it has been concluded that by conventional gravimetry and electro-gravimetry the purity of Pb was found to be 99.98 ± 0.24 and 99.97 ± 0.27 g/100 g and on the basis of Pb purity the concentration of reference standard solutions were found to be 1000.88 ± 2.44 and 1000.81 ± 2.68 mg kg^-1^ respectively with 95% confidence level (k = 2). The uncertainty evaluation has also been carried out in Pb determination following EURACHEM/GUM guidelines. The final analytical results quantifying uncertainty fulfills this requirement and gives a measure of the confidence level of the concerned laboratory.

**Conclusions:**

Gravimetry is the most reliable technique in comparison to titremetry and instrumental method and the results of gravimetry are directly traceable to SI unit. Gravimetric analysis, if methods are followed carefully, provides for exceedingly precise analysis. In classical gravimetry the major uncertainties are due to repeatability but in electro-gravimetry several other factors also affect the final results.

## Background

There is an increasing interest in Pb in the various matrixes because of its harmful effects on human beings. The Pb transfer through various processes from rocks, sediments, ground water, sludge etc. and causes negative effect on human beings. Lead as a metal has been widely used in industry for many years. Lead is omnipresent in the environment, either from natural resources or from pollutants. WHO/Codex alimentarius has established 0.01 mg L^-1^ maximum permissible limits of Pb in drinking water and 0.02 to 0.3 mg L^-1^ in food items [1]. Exposure to high concentrations of Pb can cause serious health problems, including nervous system dysfunction of fetuses and infants, hemotoxic effects, reproductive dysfunction, gastrointestinal tract alterations, nephropathies, Alzheimer’s disease, anemia etc. [2]. Thus accurate and precise determination of Pb is of prime importance because it is directly related to human health. To determine Pb at ppm or sub-ppm levels, some analytical instruments with high-sensitivity detection capabilities are available, including inductively coupled plasma mass spectroscopy (ICP-MS) and graphite furnace atomic absorption spectroscopy (GFAAS) [3-8]. Ion chromatography has also been used for lead determination; however, its sensitivity is not as good as that of ICP-MS and GFAAS [9,10]. ASTM E1613-04 is the Standard Test Method for determination of lead by ICP-AES, Flame Atomic FAAS, or GFAAS Techniques. These techniques are very sensitive and can be used only for trace analysis and always required reference standard to calibrate the instrument. The higher concentration can not be determined by these techniques due to several dilution factors. Usually the higher concentration of lead can be determined by titremetry using sodium thiosulphate, EDTA, ammonium molybdate as titrant, or electro gravimetry as PbO_2_[11] or conventional gravimetry as lead chromate, lead molybdate, lead sulphate, and lead subacetate [12]. Out of these techniques conventional gravimetry method is the primary method of measurement and most reliable technique in comparison to titremetry and instrumental method and results of gravimetry are directly traceable to SI unit.

In chemical metrology the reliability of the results in international trade is required for the acceptance of the analytical result by the users within the country or outside the country. This can be achieved by providing traceability of data characterizing products or for quality control/assurance of methodology used to analyze products. Quantifying uncertainty of analytical results fulfills this requirement and gives a measure of the confidence level of the analytical result. The estimation of uncertainty in the measurements has been given in ISO/EURACHEM guidelines [13-15]. The measurement uncertainty is carried out by quantifying of uncertainty in measurement of various steps and further by combination of potential sources of uncertainty of the entire experiment. The uncertainty has been classified as ‘Type A’, which is determined by statistical analysis of several observations. Second is or ‘Type B’ method which is determined by other ways than statistical analysis of a series of results.

Keeping in view the above facts, an attempt has been made by analyzing Pb by using two different gravimetry approaches; conventional gravimetry and electro-gravimetry. In the proposed process we have determined the purity of the Pb content with uncertainty value in the high purity lead stick procured from Sigma Aldrich. The uncertainty value determined is depends on different sources viz. uncertainty due to precision, repeatability, weighing, atomic mass of Pb and air buoyancy. In literature number of studies has been published on the evaluation of uncertainty in chemical and physical analysis [16-19] in various matrixes but the evaluation of the uncertainty by mass is very scanty. The present study highlights the various potential sources of uncertainty involved in the result obtained by conventional gravimetry and electro-gravimetry.

## Results and discussion

The gravimetric analysis only provides analysis of a single element, or a limited group of elements, at a time. In gravimetric analysis, the substance to be analyzed is separated from the other constituents in the form of an insoluble precipitate. The precipitates must be stable, non-hygroscopic and should be unaffected by the atmosphere. Besides this the precipitate should be of known chemical composition, sufficiently insoluble, easy to filter and amounts lost in the washings are insignificant. In most cases gravimetric analysis was used to determine the atomic masses of many elements up to six decimal point accuracy. It provides very less probability for instrumental error and does not require a series of standards for calculation of an unknown and also do not require expensive equipment. Due to its high degree of accuracy when performed correctly it can also be used to calibrate other instruments in lieu of reference standards. There are two basic variations of electro-gravimetric method: controlled-potential or controlled current. Electro-gravimetry analysis is more or less similar to conventional gravimetric analysis. Gravimetric analysis is one of the oldest analytical techniques and very accurate method of analysis when a large sample is available. Modern techniques require much less sample but may not be as accurate. Under the proper conditions, lead may be separated quantitatively from other metals as an insoluble sulfate. In addition, PbSO_4_ is somewhat soluble in nitric acid so there should be no nitric acid in the solution. So fuming should be strong to ensure complete removal of nitric acid from the solution. The solubility of lead sulfate increases with temperature and is dependant upon the weight percent of sulfuric acid in solution. In controlled-potential electro-gravimetry, a known constant potential is applied to the electrode for a sufficiently time to deposited out 100% of the analyte. The current decreases as the metal is deposited out. This provides a measure of selectivity if there are two or more metals that can plate out; the metal ion that is more easily reduced can often be plated out quantitatively without any of the other metal also plating out. In controlled-current electro-gravimetry, voltage is applied to the working electrode. The current is often on the order of milliamps (mA). If the concentration of metal ion in the electrolysis solution is insufficient to consume all the current, then other reactions such as hydrogen ion reduction may occur in order to consume all the current. And, if any interfering species are present, they will also plate out. In conventional gravimetry the major uncertainties are due to repeatability but in electro-gravimetry several other factors also affect the final results. After analysis of data by both the techniques, purity was found to be 99.97 ± 0.27 and 99.98 ± 0.24 g/100 g, while concentration of lead stock solutions were found to be 1000.81 ± 2.68 and 1000.88 ± 2.44 mg kg^-1^ using electro-gravimetry and conventional gravimetry technique respectively with 95% confidence level (k = 2).

## Conclusion

A generalized scheme for planning a measurement and a simple, practical approach to estimating and combining uncertainties has been demonstrated for the determination of purity of Pb stick and the concentration of Pb in stock solution by electro and conventional gravimetric techniques. The results obtained by these two methods are comparable, but standard deviation and combined uncertainty of electro-gravimetry is more in comparisons to conventional gravimetry. So on the basis of results it has been observed that conventional gravimetry is more reliable than electro-gravimetry. But before making any conclusion we should consider the limitation of the process and various sources which contribute uncertainty in final measurement. Gravimetric analysis, if methods are followed carefully, provides for exceedingly precise analysis. The proposed process can be used for the certification of purity of Pb, as traceability statement is primary requirement for the preparation of standard solutions.

### Experimental section

The first set of experiment has been carried out by electro-gravimetrically using Toshniwal electrolytic Analyzer system Model E-30, while the other set has been analyzed following classical gravimetry technique. All the weighing work has been carried out using Mettler make; AX 204 model and Sartorius make CC3002 model balances. The glasswares used were of Borosil glass works India Limited. All the acid digestion work was carried out in cleaned laminar flow bench equipped with the proper exhaustive system.

### Reagents

Nitric acid (69%), Hydrochloric acid (35%) of GR grade (Guaranteed Reagent) of Merck-India was used after purified by sub boiling point distillation of quartz glass unit. Merck make sulfuric acid (98%) GR grade was used. The Pb Sticks purity 99.999% as per certificate procured from Fluka AG, Chem (Germany), Febrik CH-9470 Buchs, 15310, Lot No; 244362587; has been used for solution preparation. De-ionized water (18.2 MΩ resistivity) prepared from Millipore milli- Q element water purification system, USA was used throughout the process.

### Lead purity determination by conventional gravimetry and electro gravimetry

Stock solution of Pb of approximately 1000 mg kg^-1^ concentration was prepared by dissolving Pb metal stick in 2 M Sub-boiled nitric acid and final volume was made to 2 kg by de-ionized water. For conventional gravimetric analysis approximately 50 g of stock solution was taken in a beaker and it was diluted to 100 mL. To this solution 4–5 ml of concentrated sulfuric acid was added. It was heated on hot plate for strong fuming for at least one hour. Then the beaker was kept in ice cold water bath after addition of 100 ml de-ionized water in to it and allowed to precipitate PbSO_4_. The precipitate was filtered through pre-weighted G-4 crucible, washed 3–4 times with 2% H_2_SO_4_ followed by de-ionized water, then cooled to the room temperature. The precipitate was dried in an oven at 110°C for 2 h there after it was cooled to the room temperature in a desiccator. The weight of the precipitate along with the G4 crucible has been taken followed by drying and cooling to the room temperature several times till a constant weight is achieved. The concentration/purity of lead was calculated by the weight difference.

**Figure 1:**
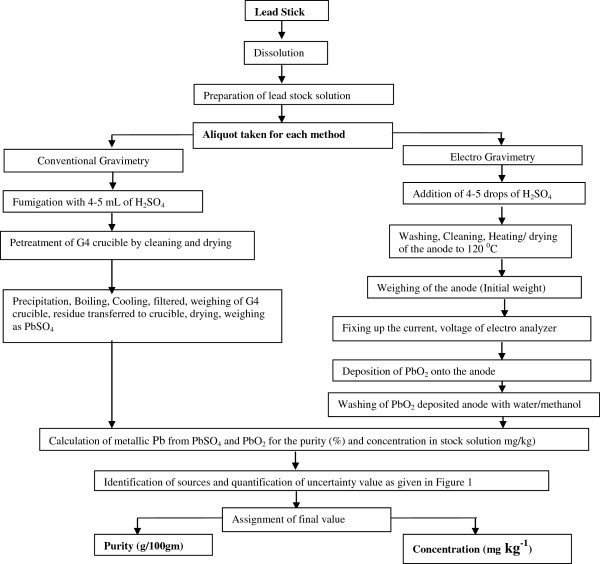
Flow chart for the purity analysis of lead by conventional and electro gravimetric method.

For electro-gravimetric analysis five replicates of approximate 50 g stock solution was taken in a beaker and it was diluted to 100 ml. To this solution 4–5 drops of concentrated sulfuric acid was added. The anode of the electro analyzer was heated at 120°C for 20–30 min. It was cooled in air and weighed. The platinum electrode was connected in the electro analyzer then the current was fixed to 0.05-0.1A at 2 V using a rheostat. The electro analyzer was kept approximate one hour to get stabilized. Further 3 to 5 ml of de-ionized water was added and anode surface was observed. The darkening of the surface of the electrode indicates the complete deposition of PbO_2_ onto it. The electrode was taken out from the beaker without stopping current. The electrode was washed thoroughly several times with de-ionized water then it was disconnected from the instrument. The anode was washed again several times with AR grade acetone and dried at 120°C for 30 min, cooled in air for 5–10 min and the final weight was taken. The concentration/purity of Pb was calculated by the difference in the initial and final weight of the electrode. The flowchart for the purity determination of Pb by conventional gravimetry and electro gravimetry has been given in Figure 1.

### Calculation of concentration in stock solution and purity of lead in lead stick

Following the EURACHEM/GUM guidelines concentration of lead *C(M*_*Pb*_*)* in the stock solution has been evaluated using gravimetry as well electro-gravimetry technique by following equation.

(1) $$C\left(M_{\mathrm{Pb}}\right)=\frac{W_{\mathrm{Pb}}F_{\mathrm{Con}}}{W_{\mathrm{samp}}}$$

Where; *C****(****M*_*Pb*_*)* = Concentration of Pb in mg/kg; W_*Pb*_ = Weight of precipitate obtained as PbO_2_ or PbSO_4_ by electro Gravimetry and conventional gravimetry respectively (g); *F*_*Con*_ = Conversion factor for PbO_2_ (0.86622) and PbSO_4_ (0.68324) in to lead and *W*_*samp*_ = Weight of sample taken for analysis. The purity of Pb in g/100g has been calculated using following equation.

(2) $$\mathrm{LeadPurity}\left(\%\right)=\frac{C{\left(M_{\mathrm{Pb}}\right)}_{\mathrm{Exp}}}{C{\left(M_{\mathrm{Pb}}\right)}_{\mathrm{Theo}}}$$

Where; *C*(*M*_*Pb*_)_*Exp*_ = Concentration of lead found by experimentally i.e. electro-gravimetry and gravimetry (mg kg^-1^); *C*(*M*_*Pb*_)_*Theo*_ = Concentration of lead found theoretically. The evaluated values for the above factors in equation 1 and 2 are given in Table 1.

**Table 1:** Various evaluated components for the determination of Lead

**Sample I.D.**	**Precipitate obtained as PbO**_**2 **_**or PbSO**_**4, **_**(W**_**Pb), **_**(g)**	**Weight of sample taken for analysis, W**_**samp, **_**(g)**	**Concentration of lead found by electro-gravimetry and gravimetry (mgkg**^**-1**^**), ( *C *(*M***_***Pb***_**)**_***Exp***_**)**	**Average concentration of lead found by electro-gravimetry and gravimetry, C**_***Pb ***_**(mg kg**^**-1**^**)**	**Theoretical Concentration of stock solution (mg kg**^**-1**^**) ( *C *(*M***_***Pb***_**)**_***Theo***_**)**	**Percent of lead analyzed by electro-gravimetry and gravimetry ( *C *(*M***_***Pb***_**)**_***Exp***_**)/ *C *(*M***_***Pb***_**)**_***Theo***_**)**	**Average (g/100 g) of lead analyzed by electro-gravimetry and gravimetry**
Pb-1	0.0584^*^	50.5550^*^	1000.641*	1000.813	100 1.1253	99.952^*^	99.969
Pb-2	0.0585*	50.6247^*^	1000.974*			99.985^*^	
Pb-3	0.0585*	50.6285^*^	1000.899*			99.977^*^	
Pb-4	0.0585*	50.6285^*^	1000.899*			99.977^*^	
Pb-5	0.0582*	50.3812^*^	1000.654*			99.953^*^	
Pb-6	0.0741**	50.5765**	1001.100**	1000.883		99.988**	99.976
Pb-7	0.0741**	50.5798**	1000.896**			99.977**	
Pb-7	0.0741**	50.5885**	1000.936**			99.981**	
Pb-9	0.0741**	50.5885**	1000.764**			99.964**	
Pb-10	0.0740**	50.5175**	1000.818**			99.969**	

* Value obtained by electro-gravimetry; ** Value obtained by conventional gravimetry.

There are several sources of uncertainty in chemical metrology like sampling, environmental conditions, uncertainties of masses, volumes, equipment, reference values, measuring equipment approximation, assumptions incorporated in experimental methods, random variations, etc. In this manuscript we have taken major contributions that are stated above in equation-1. In accordance with GUM, the combined uncertainty for the mathematical model, which is a product or quotient form, is given by:

(3) ucyy2=∑i=1Npiuxixi2

The combined standard uncertainty u_c_(y) is an estimated standard deviation and characterizes the dispersion of the values that could reasonably be attributed to the measurand ‘y’. The sensitivity coefficient = p_*i*_y/x_*i;*_ Where p_i_ is the power of the terms in the equation (1).

### Evaluation of associated uncertainty budget

The evaluation of the uncertainty of every step of the experiment is one of the requirements of the standard ISO/IEC17025 for certain test methods to get accreditation. Gravimetry analysis of Pb evaluation of combined uncertainty in such measurand is very complicated as there are various parameters to contribute uncertainty in the entire process. In the evaluation of Pb uncertainty in liquid solution there are several major sources which directly influence the final results. Out of which weighing has major contribution for adding uncertainty in the measurement of Pb due to the air buoyancy. The combined uncertainty for concentration of stock solution of lead is given by:

(4) $$u_{c}\left(C_{M_{\mathrm{Pb}}}\right)=C_{M}{}_{{}_{\mathrm{Pb}}}\sqrt[{}]{{\left[\frac{u\left(p\right)}{p}\right]}^{2}+{\left[\frac{u\left(.Pb_{\mathrm{At}.\mathrm{Wt}}\right)}{Pb_{\mathrm{At}.\mathrm{wt}.}}\right]}^{2}+{\left[\frac{u\left(B\right)}{W_{\mathrm{Metal}}}\right]}^{2}+{\left[\frac{u\left(B\right)}{W_{\mathrm{Obs}.}{}_{\;\mathrm{Stock}\;\mathrm{so}\ln.}}\right]}^{2}+{\left[\frac{u\left(\mathrm{Bu}\right)}{W_{\mathrm{True}\;\mathrm{mass}\;\mathrm{stock}\;\mathrm{so}\ln}}\right]}^{2}+{\left[\frac{u\left(B\right)}{W_{\mathrm{ppt}}}\right]}^{2}+{\left[\frac{u\left(B\right)}{W_{\mathrm{Aliquote}}}\;\right]}^{2}}$$

Similarly, the combined uncertainty for purity of lead metal is given by:

(5) $$u_{c}\left(p_{\mathrm{Pb}}\right)=p_{{}_{\mathrm{Pb}}}\sqrt[{}]{{\left[\frac{u_{c}\left(C_{M_{\mathrm{Pb}}}\right)}{C_{M}{}_{{}_{\mathrm{Pb}}}}\right]}^{2}+{\left[\frac{u\left(Pb_{\mathrm{At}.\mathrm{Wt}}\right)}{Pb_{\mathrm{At}.\mathrm{wt}.}}\right]}^{2}+{\left[\frac{u\left(B\right)}{W_{\mathrm{Metal}}}\right]}^{2}+{\left[\frac{u\left(B\right)}{W_{\mathrm{Obs}}\;{\;}_{\mathrm{Stock}\;\mathrm{so}\ln.}}\right]}^{2}+{\left[\frac{u\left(\mathrm{Bu}\right)}{W_{\mathrm{True}\;\mathrm{mass}\;\mathrm{stock}\;\mathrm{so}\ln}}\right]}^{2}+{\left[\frac{u\left(B\right)}{W_{\mathrm{ppt}}}\right]}^{2}+{\left[\frac{u\left(B\right)}{W_{\mathrm{Aliquote}}}\right]}^{2}}$$

**Figure 2:**
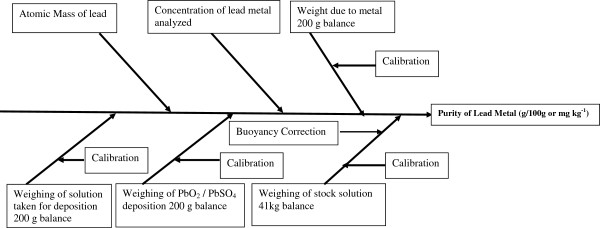
Fish- bone diagram for probable sources of uncertainty in the determination of lead by conventional gravimetry and electro gravimetry.

Where, uC is the combined uncertainty of the concentration of stock solution; *C(M*_*Pb*_*)* is the concentration of lead in mg kg^-1^ ; u(p)/p is the relative uncertainty of purity of lead metal; up is the combined uncertainty of the purity of lead; *p*_*Pb*_ is the purity of lead ; u(Cmpb)/Cmpb is the relative uncertainty of concentration of stock solution; u(Pb at. Wt)/Pb at; Wt is the relative uncertainty of atomic mass of lead ; u(B)/W_metal_ is the relative uncertainty due to weighing of lead stick; u(B)/W_stock_ solution is the relative uncertainty due to weighing of stock solution; u(Bu)/W_true_ mass is the relative uncertainty due to buoyancy correction; u(B)/Wppte. is the relative uncertainty due to weighing of precipitate; u(B)/W_aliquot_ is the relative uncertainty due to weighing of aliquot. The individual uncertainty is evaluated either by ‘Type A’ (i.e. using statistical analysis of a series of observations) or by ‘Type B’ (i.e. using other means than the statistical analysis of a series of observations). The components and sub components which contribute towards the uncertainty are shown in Fish- bone diagram (Figure 2). The uncertainty evaluation in every step of the experiment has been discussed in the following sub-sections:

### Uncertainty in purity of lead metal

**Figure 3:**
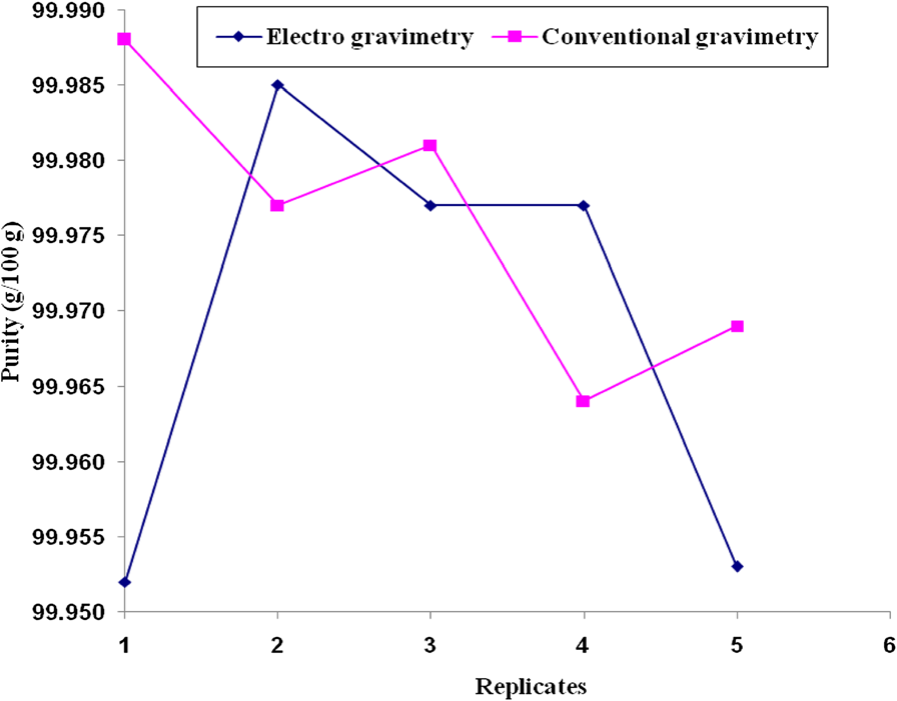
Variation of purity (g/100 g) with respect to repetition of experiments carried out by conventional gravimetry and electro gravimetry method.

**Figure 4:**
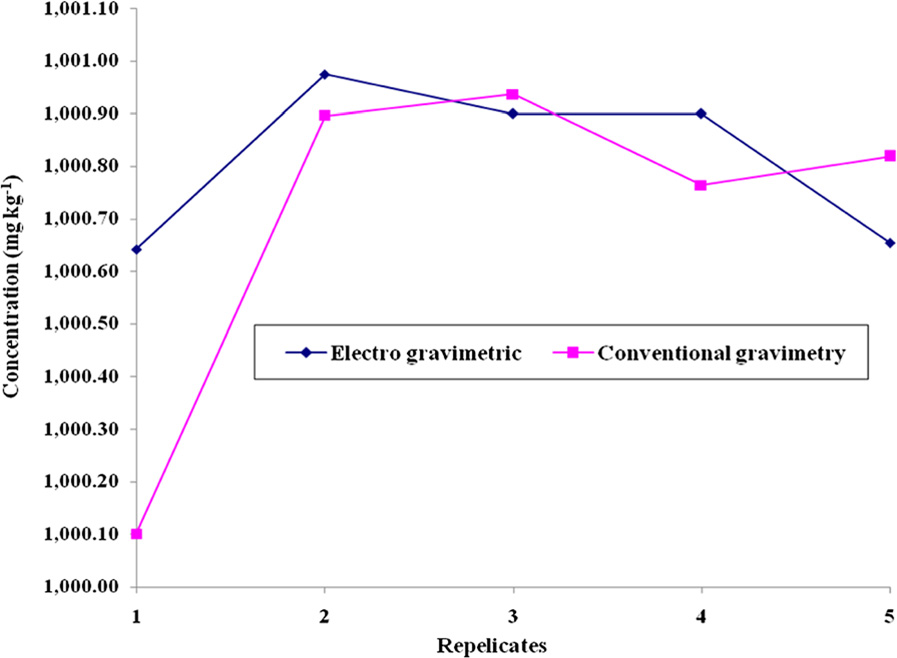
**Variation of concentration (mg kg**^**-1**^**) with respect to repetition of experiments carried out by conventional gravimetry and electro gravimetry method.**

To get the uncertainty value associated with the purity of the Pb metal, the uncertainty due to concentration obtained using equation-1 has to be considered. It could be quantified by the precipitate obtained by the repetition of experiments carried out for both the methods. The standard deviation of the measurements can be considered for the standard uncertainty with the degrees of freedom. Figures 3 and 4 shows the variation of the purity value (g/100 g) and concentration (mg kg^-1^) obtained from the five replicates. Similarly the uncertainty value associated with the concentration is given in Tables 2, 3, 4 and 5.

**Table 2:** Summary table for Uncertainty Budget for purity determination in (g/100 g) by classical gravimetry

**Sources**	**X**	**u**_***std ***_**(x)**	**u**_***std ***_**(x) / X**	**U*c *(%)**	**U**_***Ex ***_**(mg kg**^**-1**^**); k=2**
Precision	99.976%	0.0048%	0.0000476	0.122	0.244
Atomic mass of PbSO_4_	303.253 g	0.0578 g	0.000191		
Balance (Mettler 220 g)	2.015 g	0.00005 g	0.000025		
Balance (Sartorius 41 kg)	2012.734 g	0.1345 g	0.000067		
Error in buoyancy	2014.745 g	2.0012 g	0.000993		
Weight of PbSO_4_ precipitate (Mettler 220 g)	0.0741 g	0.00005 g	0.000675		
Weight of solution for deposition(Mettler 220 g)	50.5918 g	0.00005 g	9.883E-07		

**Table 3:** **Summary table for Uncertainty Budget for concentration determination in mg kg**^**-1 **^**by classical gravimetry**

**Sources**	**X**	**u**_***std ***_**(x)**	**u**_***std ***_**(x) / X**	**U*c *(mg kg**^**-1**^**)**	**U**_***Ex ***_**(mg kg**^**-1**^**); k=2**
Precision	1000.883 mg/kg	0.047 mg/kg	0.000047	1.22	2.44
Atomic mass of PbSO_4_	303.253 g	0.0578 g	0.000191		
Balance (Mettler 220 g)	2.015 g	0.00005 g	0.000025		
Balance (Saritorious 41 kg)	2012.734 g	0.1345 g	0.000067		
Error in buoyancy	2014.745 g	2.0012 g	0.000993		
Weight of PbSO_4_ precipitate (Mettler 220 g)	0.0741 g	0.00005g	0.000675		
Balance (Sartorios 41 kg) for stock solution	50.5918 g	0.00005 g	9.883E-07		

**Table 4:** Summary table for Uncertainty Budget for purity determination in (g/100g) by electro-gravimetry

**Sources**	**X**	**u**_***std ***_**(x)**	**u**_***std ***_**(x) / X**	**U*c *(%)**	**U**_***Ex ***_**(%); k=2**
Precision (P)	99.969%	0.0069%	0.00007	0.134	0.268
Atomic mass of PbO_2;_ (At. Wt)	239.199 g	0.0577 g	0.000241		
Balance (Mettler 220 g); (W_*metal*_)	2.015 g	0.00005 g	0.000025		
Balance (Sartorious 41kg) (W_*Obs. stock solution*_)	2012.734 g	0.1345 g	0.000067		
Error buoyancy; (Bu w.r.t W_*true mass stock soln*._)	2014.745 g	2.0012 g	0.000993		
weight of PbO_2_ (Mettler 220 g) (W_*ppt*_)	0.0584 g	0.00005 g	0.000856		
Weight of soln. for deposition(Mettler 220 g) (W_*aliquote*_)	50.5618 g	0.00005 g	0.000001		

**Table 5:** **Summary table for Uncertainty Budget for concentration determination in mg kg**^**-1 **^**by electro-gravimetry**

**Sources**	**X**	**u**_**std **_**(x)**	**u**_**std **_**(x) / X**	**Uc (mg/kg)**	**U**_**Ex **_**(mg kg**^**-1**^**);k=2**
Precision (C_MPb_)	1000.813 mg/kg	0.069 mg/kg	0.000069	1.338	2.676
Atomic mass of PbO_2_	239.199 g	0.0577 g	0.000241		
Balance (Mettler 220 g)	2.015 g	0.00005 g	0.000025		
Balance (Sartorious 41 kg)	2012.734 g	0.1345 g	0.000067		
Error in buoyancy	2014.745 g	2.0012 g	0.000993		
weight of PbO_2_ (Mettler 220 g)	0.0584 g	0.00005 g	0.000856		
Weight of solution taken for electro-gravimetry experiment (Mettler 220 g)	50.5618 g	0.00005 g	0.000001		

### Uncertainty in atomic mass

Atomic mass of the element with uncertainty is obtained from the IUPAC Technical report [20]. As lead obtained as PbO_2_ and PbSO_4_ by weight, hence the atomic mass of O & S are also taken into consideration in the uncertainty calculation. To obtain the standard uncertainty due to atomic mass the IUPAC quoted uncertainty considers a rectangular distribution as given in the Table 6.

**Table 6:** Uncertainty in atomic mass of lead Metal

**Element**	**Atomic mass of lead (g)**	**Uncertainty in weight (g)**	**Distribution**	**Standard uncertainty**	**Combined standard uncertainty**
Pb	207.19	0.1	Rectangular	0.1/√3=0.05773	0.0578 (PbSO_4_)
S	32.065	0.005	Rectangular	0.005/√3=0.00289^#^	
O^#^	15.9994	0.0003	Rectangular	0.0003/√3=0.00017^#^ =0.00017*4=0.00068	
O^#^	15.9994	0.0003	Rectangular	0.0003/√3=0.00017^#^ =0.00017*2=0.00034	0.0577 (PbO)

In gravimetry lead obtained in the form of PbSO_4_/ PbO_2;_

O^#^ four atoms of oxygen have been considered in combined uncertainty for PbSO_4_ and two atoms have been considered for PbO.

### Uncertainty in weighing

There are several factors such as repeatability, nonlinearity, sensitivity, air buoyancy, which influence to the weight of the material while weighing in an electronic balance. There are two types of balances have been used for the whole experiment as given in the section 2 of this paper. The initial weight of approximately 2 g Pb stick was taken in a micro balance and the stock metal solution weight taken in a balance of 41 kg**.** The amount of stock solution taken for deposition of Pb and the weight of the precipitate obtained after conventional gravimetry method as well as electrolytic deposition on electrode are also taken in micro balance. The uncertainty associated due to calibration of the balance is taken from the calibration certificate provided by CSIR-National Physical Laboratory, New Delhi, National metrology institute of India and are given in Table 7. But in the case of the preparation of 2 kg weight solution, the correction due to air buoyancy has been included as the weight taken in presence of air influences much in the observed weight. The reference regarding calculation of buoyancy correction is very scanty. The EURACHEM/CITAC Guide CG 4 is also not elaborating the uncertainty in weighing properly. In this paper effort has been taken to calculate the true weight considering the buoyancy correction.

**Table 7:** Uncertainty due to balance of Ohaus make (200 g) and Sartorius make (41 kg)

**Weight component**	**Balance used for weighing**	**Weight taken (g)**	**Uncertainty in weight as per certificate at k=2**	**Distribution**	**Standard uncertainty in total weight u(x)/x**
Weight of the metal m(g)	Mettler AX 204 220g	2.015	0.0001	Normal	0.0001/2=0.00005
Weight of solution taken for PbO_2_ deposition	do	50.5618	0.0001	Normal	0.0001/2=0.00005
Weight of PbO_2_ deposited	do	0.0584	0.0001	Normal	0.0001/2=0.00005
Weight of solution taken for PbSO_4_ deposition	do	50.5918	0.0001	Normal	0.0001/2=0.00005
Weight of PbSO_4_ deposited	do	0.0741	0.0001	Normal	0.0001/2=0.00005
Weight of stock solution	Sartorius; CC3002; 41 kg	2012.734	0.269	Normal	0.269/2=0.1345

### Calculation of buoyancy correction

In the proposed process uncertainty due to buoyancy has been consider first time, which has major effects on weighing. The mass of the solution taken in an electronic balance is actually measure its weight force. So in presence of air the weight taken in an electronic balance varies remarkably for the same sample. As there is a deviation in the weight taken in an electronic balance due to air buoyancy, so the total uncertainty budget by mass is calculated considering buoyancy correction. The EURACHEM/CITAC Guide CG 4 [21]; Jones, Schoonover and Gonz´alez [22-24] have presented the process to calculate correction due to air buoyancy. For the calibration of single-pan electronic control balance, a standard weight of known mass has to be used to builds an apparent mass calibration into the balance. The reference density of the apparent mass scale is the density of the standard mass used for the calibration and the reference air density is the air density at the time of calibration.

Air density can be calculated using the following equation.

(6) $$\rho_{a}=\frac{0.34848\times P-0.009\times \mathrm{RH}\times \exp\left(0.062\times T\right)}{\left(273.154+T\right)}$$

Putting the following values of temperature, pressure and humidity in the above equation. The weight of an unknown sample weighed on the balance is then

(7) $$W_{T}=\frac{W_{\mathrm{Obs}}}{\left(1-\rho_{a}M/\rho_{{}_{S}}\right)}\times \left(1-\rho_{a}/\rho_{{}_{X}}\right)$$

Where; W_*obs*_ is the observed weight of the unknown sample; W_*T*_ is the True weight of the unknown sample; ρ_*a*_ is the density of air at the time of balance calibration; ρ_*a*_M is the density of air at the time of measurement; ρ_*x*_ is the density of the standard used to calibrate the balance; ρ_*s*_ is the density of the sample. It is shown that the relative error in neglecting buoyancy is about 0.1 %. So, the exact value of the error is easily calculated as follows

W_*obs*_ = W_*T*_ × F; where, $F=\frac{\left(1-\rho_{a}M/\rho_{S}\right)}{\left(1-\rho_{a}/\rho_{x}\right)}$

In this case ρ_s_ is the density of the elemental solution which is nothing but 2% acidified de-ionized water. Putting the value of ‘F’ and W_*obs*_ in equation −5, so true weight can be calculated. The true weight of the stock solution is found to be 2014.745 g. The relative percent error in neglecting buoyancy is given by

(8) $$\left(W_{T}-W_{\text{obs}}\right)\;100\;/\;W_{T}\;=\;100\;(1-F)$$

The absolute error (Abs_*er*_) is given by

Abs_*er*_ = W_*T*_ (1 – F)

Putting the value of W_T_ & F in Eq. (6)

Absolute error due to Buoyancy is calculated as 2.0012 g

So the relative error is u(Abs_*er*_)/ W_*T*_ = 0.000993.

The uncertainties associated with each component are combined and expanded Uncertainty with 95% confidence level.

### Calculation of combined uncertainty

**Figure 5:**
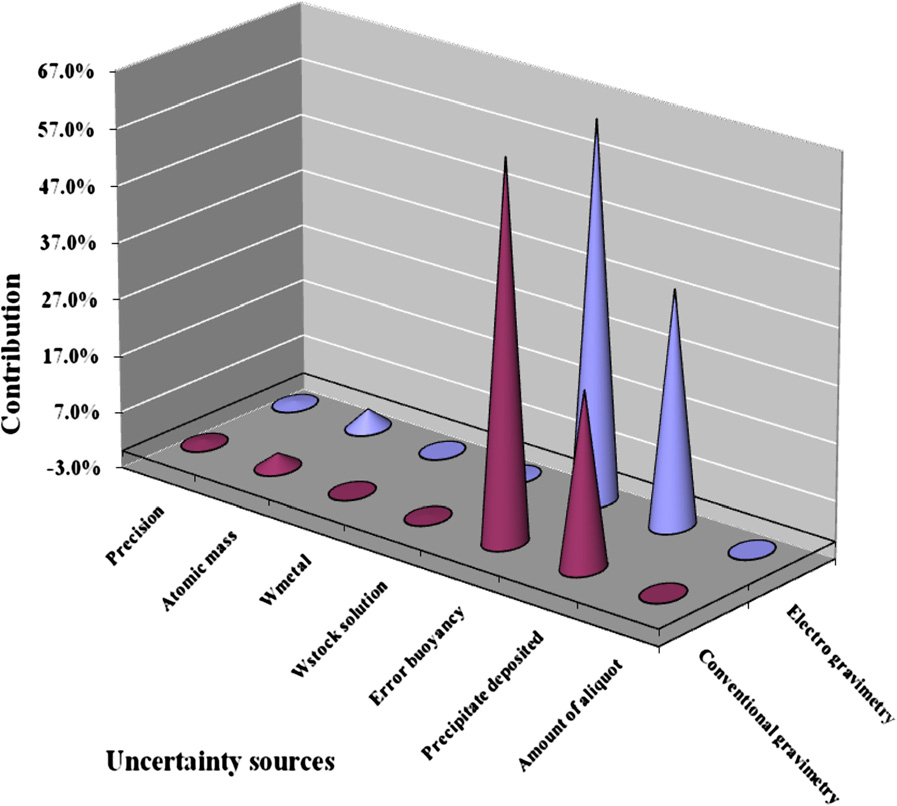
Percentage contribution of uncertainty source included in the lead determination by conventional gravimetry and electro gravimetry.

The concentration of lead (mg kg^-1^) and purity (g/100g) in five replicates following electro-gravimetry and conventional gravimetry has been determined using equation-1 and 2 respectively which is described in section 5.1 Substituting the values from Table 1 into eqution-1 and 2, the concentration and purity of lead respectively has been calculated for each experiment. The combined uncertainties in the determination of both concentration and purity of lead for electro-gravimetry and conventional gravimetry have been calculated by putting the value in eqution-3 and 4. The expanded uncertainty was calculated at 95% confidence level at k = 2. The percentage contribution of each uncertainty source considered in the total uncertainty budget is plotted for both gravimetric methods (Figure 5). It shows that the maximum contribution of uncertainty lies due to the weighing of precipitate of PbO_2_, PbSO_4_ and the stock lead solution. However, the associated uncertainty value obtained for the electro gravimetric method is more as compared to the conventional gravimetric method due to the uncertainty due to atomic mass and the precipitated deposited sources.

## Competing interests

The authors declare that they have no competing interests.

## Authors’ contributions

Nahar made a significant contribution to acquisition of data, analysis and manuscript preparation. Experiential work has been carried out by Nahar and Niranjan. SST has done theoretical calculation of buoyancy correction. KS and DS and PKG made a significant contribution experimental design and data analysis. All the authors read and approved the final manuscript
